# The course of physical functional limitations and occupational conditions in a middle-aged working population in France

**DOI:** 10.1186/1745-6673-7-5

**Published:** 2012-04-11

**Authors:** Matthieu de Stampa, Aurélien Latouche, Francis Derriennic, Christine Monfort, Annie Touranchet, Bernard Cassou

**Affiliations:** 1 UPRES EA 2506 Laboratoire Santé-Environnement-Vieillissement, AP-HP, Hôpital Sainte Perine, Université Versailles St-Quentin, Paris, France; 2 INSERM, Unité 754, 16 Avenue Paul-Vaillant Couturier, 94807, Villejuif, France; 3 Inspection médicale régionale du travail des Pays de Loire, 26 Boulevard Vincent Gâche, 44063, Nantes, Cedex 02, France; 4 UPRES EA 2506 Laboratoire Santé-Environnement-Vieillissement, AP-HP, Hôpital Sainte Perine, Université Versailles St-Quentin, 49 rue Mirabeau, 75016, Paris, France

**Keywords:** Physical functional limitations, Physical occupational factors, Psychosocial occupational factors, Working population

## Abstract

**Background:**

Physical functional limitations (PFL) have mainly been studied in older populations. The aim of this study was to better understand the course of PFL and associations with occupational factors by gender in a middle-aged working population.

**Methods:**

The data came from 16,950 workers in the ESTEV (Enquête Santé Travail et Vieillissement) cohort in France. PFL were assessed using the physical abilities section of the Nottingham Health Profile. Occupational conditions were measured with a self-administered questionnaire covering physical and psychosocial factors in 1990 and 1995. Multivariate analyses were used to assess the associations.

**Results:**

The PFL appearance rate in 1995 was the same by gender (6.3%); the rate of PFL recovery was higher in men (23.9% versus 20.9%). Age was an independent factor of PFL at age 47 years or older in both genders after adjusting for confounding factors. The PFL appearance rate in 1995 was higher with physical occupational exposure in 1990, such as awkward work with a dose relation in both genders, while the PFL recovery rate decreased significantly only for men. Exposure to psychosocial occupational conditions, such as having the means to produce quality work in 1990, was significantly associated with a decreased PFL appearance rate in 1995 in both genders, and having high decision latitude in 1990 was associated with a decreased PFL appearance rate in 1995 only in men. Changes in exposure to occupational factors between 1990 and 1995 were associated with the PFL appearance and recovery rates in 1995 in both genders.

**Conclusions:**

After five years, the course of PFL in this working population changed and was associated with physical and psychosocial occupational factors. Relationships were stronger for the PFL appearance rate in both genders and were weaker for recovery from PFL, mainly among women.

## Background

Many governments are encouraging workers and, in particular, the oldest workers, to stay employed for longer periods [1]. But work-related musculoskeletal disorders (MSD) are a leading cause of morbidity in the European Union [2]. Pain at multiple sites is a common phenomenon among working people and is strongly associated with self-rated poor physical work ability [3]. According to an epidemiological surveillance system for work-related MSD implemented in 2002 in France’s Pays de la Loire region, 11% of men and 15% of women had at least one of six main upper-limb clinically-diagnosed MSD [4]. In 2004, over 25,000 workers were awarded workers’ compensation for limb MSD by France’s general National Health Insurance Fund [4].

Moreover, MSD are the main cause of disability before the age of 45 and rank first among the causes of health-related work limitations [5,6]. One of the first manifestations of disability is physical functional limitations (PFL) [7], which can lead to sick leave and early retirement [8,9]. PFL are the functional consequence of illnesses mainly related to MSD and increase with the number of morbidities [10]. PFL are defined according to the individual’s difficulty or inability to perform daily activities such as walking, climbing and lifting objects [11]. Some workers with MSD become disabled, while others do not [12]. Being a woman may be associated with a higher rate of sick leaves [13] and disability leaves due to MSD [14]. The individual course of MSD is not stable, and may fluctuate over time in response to improvement and worsening factors [15].

Although there is growing evidence that physical and psychological occupational factors increase the risk of MSD and are related to their course [16], it is not clear whether these factors influence PFL in the same way. PFL have mainly been studied in older populations, and little is known about their course in the working population and paths by gender, which are often neglected. In the workplace, preventive measures have primarily been focused on MSD, so data about PFL could influence preventive strategies designed to reduce the appearance of PFL or improve PFL recovery rates. The objectives of this study are therefore to describe the course of PFL in a middle-aged working population and to assess relationships with occupational conditions by gender. It is essential to determine the associations between the work factors and PFL for implementing preventive actions. We used data from ESTEV (Enquête Santé Travail et Vieillissement), a French prospective study whose large representative sample of workers allowed us to associate PFL appearance and recovery rates with changes in occupational conditions over a five-year period.

## Methods

The data came from the French ESTEV study, a prospective longitudinal epidemiological investigation designed to identify occupational factors that may modify the development of a variety of health characteristics. The population and methods of this study have previously been described in detail [17].

### Selection of participants

The sample was randomly selected in 1990 from exhaustive lists of workers in the private sector who saw occupational physicians for legally required annual examinations. These physicians were recruited through regional associations that covered seven socioeconomically contrasting regions of France (Bretagne, Dauphiné, Ile de France, Nord, Pays de Loire, Val de Loire and Ile de la Reunion). In all, 387 physicians (approximately 60% of those initially approached) volunteered to participate. The survey took place during compulsory annual visits to an occupational physician in 1990 and 1995. To identify age-related inter-individual variations in health and generational effects, the ESTEV study focused on four specific cohorts representing four generations: individuals born in 1938, 1943, 1948 and 1953. Each occupational physician classified patients in eight categories according to sex and year of birth and then used a specific sampling coefficient to randomly select an equal number of participants from each file for the sample. National data on the distribution of French wage-earners according to age and sex were used to define these coefficients. In 1990, 24,228 workers were selected, and the participation rate was 88.2%, with 21,369 participants (58.2% men). The participation rate did not vary by age, gender, or region. In 1995, 18,695 participants (87.4% of the initial sample) were assessed again; 90.6% of them (n=16,950) were still working. The 1,745 participants who were not working in 1995 were unemployed (n=732), retired (n=611), or on sick leave (n=402). Participants who were dropped from the study reported PFL more often in 1990 (+10.5%). Our study sample thus comprised the 16,950 working participants who were seen again five years after the first interview: 7,163 women (42.3%) and 9,787 men (57.7%`). The study was approved by the local committee for the protection of individuals. Workers agreeing to participate in the study were free to leave it at any time.

### Instruments

The same self-administered questionnaire on perceived health, derived from the Nottingham Health Profile (NHP) [18], was used to assess PFL in 1990 and 1995. We used the seven dichotomous (yes/no) questions on physical ability to evaluate PFL. These questions are used to assess an individual’s degree of capacity in activities of daily living and are based on upper and lower limb movements. The two questions on upper limb movements were: “I have difficulty getting dressed and undressed” and “I have difficulty reaching for objects with my arm.” For lower limb movements, the three questions were: “I can only walk about inside (home or building),” “I have problems going up and down stairs,” and “I am completely unable to walk.” For back-related functional limitations, the two questions were: “I have difficulty standing up for any length of time” and “I have trouble leaning forward (to tie my shoes, pick something up, etc.).” Workers were considered to have PFL when they reported two or more of these seven difficulties (PFL2+) in either 1990 or 1995 or both. Workers were considered PFL-free when they reported no difficulty (PFL0) or only one difficulty (PFL1).

We used the same questionnaire in 1990 and 1995, including 30 closed-ended questions, to assess exposure to physical and psychosocial work factors. The questionnaire was completed by the workers themselves during their annual occupational medical examination and was checked by the occupational physician during this assessment. This questionnaire was designed on the basis of previous epidemiological and ergonomic research conducted in France [19]. A validation study of approximately 200 workers verified that the questions were understandable.

Physically awkward work was assessed with data on five factors collected using dichotomous questions (yes/no): “Do you work in awkward postures, carry heavy loads, or have to walk frequently?” “Are you exposed to vibrations?” and “Is exertion required to operate tools or machines?” This physically awkward work was categorized as: not exposed, exposed to one factor, or exposed to two or more factors. Precision work requiring concentration was assessed using three criteria: the need to detect fine details, execute precise motor actions and not take one’s eyes off or interrupt one’s work. This working condition was categorized as either not exposed or as exposed to at least one factor. Repetitive work under time constraints was determined using the subject’s dichotomous self-assessment. An assessment of non-standard working hours was constructed from five factors: work exceeding 48 hours per week, work in rotating or staggered shifts, work schedules or travel times that often require going to bed after midnight, rising before 5:00 a.m., or missing a night’s sleep (at least 50 days of the year). The variable of non-standard working hours was categorized as either not exposed or exposed to at least one of these factors.

Latitude in decision making and psychological pressure were constructed as proxies for dimensions of Karasek’s “job demands–job autonomy” model [20]. More specifically, latitude in decision making referred to the worker’s autonomy and was assessed using three dichotomous factors: varied work, work that provides learning opportunities, and some choices in how to proceed with the work. This psychosocial factor was categorized in terms of levels of exposure as either low (not exposed or exposed to one factor) or high (at least two factors). Psychological pressure was assessed using three factors: frequent need to rush, need to do several things at the same time, and frequent interruption of one’s work by others. Psychological pressure was categorized as either low exposure (not exposed or exposed to one factor) or high exposure (at least two factors).

Finally, workers were asked if they had the means to produce quality work, i.e. if they had the necessary materials, information and time for such work.

Potential confounding factors were selected using social, physical activity, and mental health data. The social data included French socio-professional categories (SPCs), gender and year of birth. SPCs were derived from the French system of classification used by INSEE (France’s Institute of Statistics and Economic Studies) and consisted of four groups: management and intellectual professions (Group 1); intermediate professions, including skilled tradespeople, merchants, administrative and commercial professions, technicians, foremen and line supervisors (Group 2); office and sales workers (Group 3); and qualified or unqualified blue-collar workers (Group 4). The categories were combined into two groups: “managers” (Groups 1 and 2) and “non-managers” (Groups 3 and 4). Physical activities included sporting activities and were categorized as exposed or not exposed as declared by the participant. Mental health data identified the presence of anxiety and/or depressive disorders based on the NHP’s “emotional reactions.” The assessment involved nine factors: becoming increasingly discouraged, realizing that nothing was good enough anymore, feeling nervous and tense, finding the day very long, easily getting angry, having difficulty dealing with what was happening, having concerns that were inferring with sleep, feeling that life was not worth living, and waking up depressed. The factor “emotional reactions” was categorized as not exposed or exposed to at least one factor.

### Statistical analyses

PFL appearance and recovery rates in 1995 were analyzed according to exposure to occupational factors in 1990 in both genders. Multivariate analyses took confounding factors into account to explain relationships between appearance of and recovery from PFL and work-related factors. The analyses were performed by logistic regression, adding or removing explanatory variables from the model using an ascending stepwise procedure with the significance level set at 10%. The same procedures were used to test interactions between variables.

PFL appearance and recovery rates in 1995 were analyzed according to changes in working conditions between 1990 and 1995 in both genders. The presence (E+) or absence (E-) of specific occupational conditions in 1990 and 1995 were used to classify participants into one of four course typologies: E-E-; E-E+, E+E+; E+E-. Associations were tested using a Chi-square test and quantified using the odds ratio (OR) and a 95% confidence interval. Results were adjusted for age and stratified by gender.

The data were analyzed using SAS software for descriptive analyses and BMDP software for logistic regressions on a UNIX server at the INSERM computer center.

## Results

In 1990, the study sample consisted primarily of non-managers, particularly among the women (see Table 1). Men were exposed more often to physically awkward work, and women to repetitive work. Exposure to non-standard hours was more frequent among men and increased with age. Decision latitude and having the means to produce quality work were high in both genders, and psychological pressures were identified for over 56% of the participants.

**Table 1:** Baseline characteristics of workers by gender and birth year cohort in 1990 (n=16, 950)

	**Women**					**Men**				
	**Total**	**1953**	**1948**	**1943**	**1938**	**Total**	**1953**	**1948**	**1943**	**1938**
N	7163	1924	1893	1976	1370	9787	2639	2768	2665	1715
%	42.3	42.1	40.6	42.7	44.4	57.7	57.8	59.3	57.4	55.8
Non-managers (%)	77.5	76.7	77.1	76.9	80.5	64.3	68.1	64.2	60.7	64.6
**Physical factors**										
Awkward work (%)	54.1	48.8	52.9	54.8	61.7	76.5	75.9	75.3	76.3	79.7
1 factor	21.6	20.9	21.1	21.8	23.5	20.6	21.1	20.3	21	19.9
2 factors	17.2	15.2	16.9	17.9	19.6	21.3	20.8	21.3	20.7	23
> 2 factors	15.3	12.7	14.9	15.1	18.6	34.6	34	33.7	34.6	36.8
Precision work (%)	42.8	40.1	42.5	41.7	46.3	47.4	46.1	47.8	48.4	46.6
Repetitive work (%)	21.7	20.8	22.7	22.4	20.7	17.8	18.8	18.2	17	16.9
Non-standard hours (%)	43.9	35	43.7	46.5	53	78.2	71.1	77.9	81	85.8
**Psychosocial factors**										
High latitude (%)	77.5	78.4	78.7	78.1	74	86.5	92	86.2	85.7	80.3
High pressure (%)	56.6	59.2	56.1	57.2	52.9	55.6	54.7	57.1	57.5	51.8
Means for quality (%)	75.6	73.7	75.6	76	77.5	78.8	76.7	78.2	80.2	80.8

Birth year cohort with participants born in 1953, 1948, 1943, 1938.

In 1990, cases of PFL2+ were more frequent among women (13.1%, compared with 11.6% among men, p<0.01) (see Table 2). The appearance of PFL2+, defined as the number of workers newly affected in 1995 divided by the number unaffected in 1990 (PLF0), was similar for men and women: 6.3% of workers. The recovery rate, defined as the number of workers affected in 1990 (PLF2+) who were unaffected in 1995 (PLF0), was higher but not significant among men (23.9%) compared to women (20.9%). Over 50% of women and men reported no PFL in both 1990 and 1995. Conversely, 7.4% of women and 6.4% of men reported PFL in both 1990 and 1995.

**Table 2:** Prevalence of PFL in 1990 and PFL appearance and recovery rates in 1995 by gender (n=16,950)

	**WOMEN (n=7,163)**			**MEN (n=9,787)**			
	**PFL0 in 1995 n (%)**	**PFL1 in 1995 n (%)**	**PFL2+ in 1995 n (%)**		**PFL0 in 1995 n (%)**	**PFL1 in 1995 n (%)**	**PFL2+ in1995 n (%)**
**PFL0 in 1990** 4,620 (64.5%)	3,640 (78.8)	688 (14.9)	292 (6.3)	**PFL0 in 1990** 6,587 (67.3%)	5,368 (81.5)	804 (12.2)	415 (6.3)
**PFL1 in 1990** 1,602 (22.4%)	711 (44.4)	546 (34.1)	345 (21.5)	**PFL1 in 1990** 2,065 (21.1%)	968 (46.9)	634 (30.6)	463 (22.5)
**PFL2+in 1990** 941 (13.1%)*	197 (20.9)	214 (22.8)	530 (56.3)	**PFL2+ in 1990** 1,135 (11.6%)	271 (23.9)	241 (21.1)	623 (54.9)

PFL = physical functional limitations.

PFL0 = no physical difficulties with upper limbs, lower limbs, or spine.

PFL1 = one physical difficulty with upper limbs, lower limbs, or spine.

PFL2+ = PFL = two or more physical difficulties with upper limb, lower limb, or spine problems.

*p<0.001.

Age was independently associated with a higher occurrence of PFL in both genders born in 1943 and 1938 (47 years old and over) when adjusted for confounding factors in an ascending stepwise procedure (see Table 3). The PFL appearance rate in 1995 was higher with exposure to awkward work in 1990 in both genders with a dose relationship of two factors or more. Exposure to psychosocial occupational conditions such as having the means to produce quality work in 1990 were significantly associated with a decreased PFL appearance rate in 1995, and having high latitude in decision making was associated with a decreased PFL rate only in men. The PFL recovery rate decreased significantly with exposure to awkward work only in men. Emotional reactions were associated with an increased PFL rate, and sporting activities were associated with a decreased PFL rate in both genders. No interactions were found between factors.

**Table 3:** PFL appearance and recovery rates in 1995 and occupational exposure factors in 1990 by gender (Logistic Regression)

	**Appearance of PFL**				**Recovery from PFL**			
	**Women**		**Men**		**Women**		**Men**	
	**Order**	**OR (95% CI)**	**Order**	**OR (95% CI)**	**Order**	**OR (95% CI)**	**Order**	**OR (95% CI)**
**Emotional reactions**	1		1		2		3	
No		1		1		1		1
Yes		1.6 (1.4–1.9)		1.75(1.5–2.0)		0.7 (0.5–0.9)		0.8 (0.7–0.9)
**Age (born in)**	2		3		1		1	
1953		1		1		1		1
1948		1.1 (0.9–1.4)		1.0 (0.9–1.2)		0.7 (0.6–0.9)		0.9 (0.7–1.2)
1943		1.6 (1.3–1.9)		1.4 (1.2–1.6)		0.5 (0.4–0.7)		0.9 (0.7–1.1)
1938		1.7 (1.4–2.1)		1.8 (1.5–2.1)		0.4 (0.3–0.6)		0.6 (0.5–0.8)
**Sporting activities**	3		4		3		4	
No		1		1		1		1
Yes		0.7 (0.6–0.8)		0.85 (0.7–0.95)		1.3 (1.1–1.5)		1.2 (1.0–1.5)
**Awkward work**	4		2		//		2	
No factors		1		1				1
1 factor		0.9 (0.7–1.1)		1.2 (0.9–1.5)				0.9 (0.7–1.2)
2 factors		1.3 (1.1–1.5)		1.4 (1.2–1.8)				0.9 (0.7–1.1)
>2 Factors		1.4 (1.1–1.6)		1.7 (1.3–2.0)				0.7 (0.5–0.9)
**Having means**	5		6		//		//	
No		1		1				
Yes		0.7 (0.6–0.8)		0.7 (0.6–0.8)				
**High decision latitude**	//		5		//		//	
No				1				
Yes				0.7 (0.6–0.8)				

PFL = two and more difficulties in physical abilities from upper limb, lower limb or spine problems.

Order: order that factors were entered into the logistic regression model using an ascending stepwise procedure.

OR: Odds Ratio.

CI 95%: Confidence Interval 95%.

Adjusted for age, physical factors (awkward work, precise, repetitive work, non-standard hours), psychosocial factors (high decision latitude, psychological pressure, having means), sporting activities, emotional reactions.

//: Factors not selected by the model.

Among workers not exposed in 1990 and exposed in 1995 (E-E+) compared to non-exposure at both times (E-E-), the PFL appearance rate in 1995 increased in both genders exposed to physical occupational factors such as awkward work, repetitive work and non-standard hours (see Table 4). The risk was higher for awkward work for men and repetitive work for women. Men exposed to repetitive work in 1995 and not exposed in 1990 were less likely to report recovery from PFL in 1995. Concerning exposures to psychosocial occupational conditions, the PFL appearance rate in 1995 had decreased significantly in both genders for workers having the means to produce quality work in 1995 but not in 1990 when compared to those who were not exposed in 1990 and 1995.

**Table 4:** PFL appearance and recovery rates in 1995 with absence in 1990 and presence in 1995 of physical and psychosocial occupational factors (E-E+) compared to non exposure at both times (E-E-), by gender and adjusted for age

	**Appearance of PFL**				**Recovery from PFL**			
	**Women**		**Men**		**Women**		**Men**	
	**N**	**OR CI 95%**	**N**	**OR CI 95%**	**N**	**OR CI 95%**	**N**	**OR CI 95%**
**Physical factors**								
**Awkward work**								
E-E-	1568	1	1291	1	964	1	345	1
E-E+	468	1.2 (0.8 - 1.9)	507	2.6 (1.5 to 3.7)	197	0.8 (0.4 - 1.1)	157	0.8 (0.5 - 1.1)
**Precise work**								
E-E-	2151	1	3633	1	1129	1	567	1
E-E+	483	1.1 (0.7 - 1.6)	746	1.1 (0.8 - 1.4)	272	0.9 (0.6 - 1.9)	181	0.9 (0.6 - 1.7)
**Repetitive work**								
E-E-	3497	1	5619	1	1615	1	1855	1
E-E+	273	2.6 (1.8 - 3.9)	417	1.8 (1.3 - 2.5)	201	0.9 (0.5 - 1.3)	148	0.7 (0.4 – 0.9)
**Non standard hours**								
E-E-	3218	1	1068	1	545	1	365	1
E-E+	126	1.9 (1.3 - 2.8)	552	1.7 (1.2 - 2.4)	115	1.1 (0.6 - 1.6)	147	0.9 (0.6 - 1.5)
**Psychosocial factors**								
**High decision latitude**								
E-E-	529	1	425	1	404	1	330	1
E-E+	431	0.9 (0.6 - 1.5)	318	0.9 (0.7 - 1.1)	247	1.1 (0.7 - 1.6)	248	1.3 (0.7 - 2.5)
**Psychological pressure**								
E-E-	1386	1	2316	1	676	1	761	1
E-E+	673	0.9 (0.6 - 1.3)	948	1.2 (0.8 - 1.5)	356	1.0 (0.6 - 1.4)	318	0.8 (0.6 - 1.2)
**Having means**								
E-E-	454	1	558	1	313	1	265	1
E-E+	609	0.6 (0.4 - 0.9)	819	0.6 (0.4 - 0.9)	366	1.2 (0.7 - 1.6)	422	1.4 (0.8 - 2.1)

OR : Odds Ratio.

CI 95% : Confidence Interval 95%.

PFL : two and more difficulties in physical abilities from upper limb, lower limb or spine problems.

Among workers of both genders exposed in 1990 but not exposed in 1995 (E+E-) as compared to exposure at both times (E+E+), the awkward work factor was associated with a decreased PFL appearance rate in both genders (see Table 5). Women exposed to non-standard hours in 1990 and not exposed in 1995 were more likely to report recovery from PFL in 1995. For the psychosocial occupational conditions, the PFL appearance rate in 1995 increased significantly in both genders among workers who reported high decision latitude and having the means to produce quality work in 1990 but not in 1995 compared to those who were exposed in 1990 and 1995. Men with high decision latitude in 1990 but not in 1995 were less likely to report recovery from PFL in 1995. There was no interaction between PFL and occupational factors by age and sex (results not shown).

**Table 5:** PFL appearance and recovery rates in 1995 with presence in 1990 and absence in 1995 of physical and psychosocial occupational factors (E+E-) compared to exposure at both times (E+E+), by gender and adjusted for age

	**Appearance of PFL**				**Recovery from PFL**			
	**Women**		**Men**		**Women**		**Men**	
	**N**	**OR CI 95%**	**N**	**OR CI 95%**	**N**	**OR CI 95%**	**N**	**OR CI 95%**
**Physical factors**								
**Awkward work**								
E+E+	1672	1	4601	1	1219	1	1899	1
E+E-	566	0.4 (0.3 – 0.7)	771	0.4 (0.3 - 0.6)	311	1.2 (0.5 - 1.8)	212	1.3 (0.8 – 2.1)
**Precise work**								
E+E+	824	1	2314	1	1201	1	1337	1
E+E-	511	09 (0.7 - 1.3)	655	1.1 (0.8 - 1.4)	482	1.0 (0.6 - 1.6)	314	1.1 (0.6 - 1.6)
**Repetitive work**								
E+E+	485	1	695	1	731	1	354	1
E+E-	208	0.7 (0.4 - 1.1)	451	0.7 (1.5 - 1.1)	124	1.1 (0.6 - 1.9)	243	1.3 (0.7 – 2.0)
**Non standard hours**								
E+E+	1338	1	4568	1	807	1	1673	1
E+E-	651	0.9 (0.6 - 1.3)	1065	0.9 (0.6 - 1.3)	340	1.6 (1.1 - 2.4)	342	1.2 (0.8 - 1.6)
**Psychosocial factors**								
**High decision latitude**								
E+E+	3276	1	5572	1	1444	1	1992	1
E+E-	564	2.0 (1.4 - 2.7)	649	2.0 (1.5 - 2.6)	253	0.8 (0.5 - 1.4)	247	0.5 (0.3 - 0.8)
**Psychological pressure**								
E+E+	2063	1	3044	1	1014	1	951	1
E+E-	632	1.0 (0.6 - 1.4)	1034	1.2 (0.8 - 1.5)	322	1.0 (0.7 - 1.4)	399	0.9 (0.7 - 1.5)
**Having means**								
E+E+	3117	1	5016	1	1312	1	1609	1
E+E-	597	1.5 (1.1 - 2.0)	765	1.8 (1.2 - 2.1)	363	0.9 (0.7 - 1.3)	280	0.8 (0.5 - 1.2)

OR: Odds Ratio.

CI 95%: Confidence Interval 95%.

PFL: two and more difficulties in physical abilities from upper limb, lower limb or spine problems.

## Discussion

In this large middle-aged working population, the course of PFL changed over a 5-year period and the appearance rate was lower than the recovery rate for both genders. Associations between PFL and occupational conditions were stronger for the appearance rate in both genders and weaker for the recovery rate, mainly among women. Physical occupational factors were associated with an increased PFL appearance rate in both genders, mainly for the onset of exposure between 1990 and 1995. Psychosocial occupational factors were associated with a decreased PFL appearance rate and the association was stronger for the extinction of exposure between 1990 and 1995 for both genders.

The PFL rate was lower than that for MSD assessed in the same sample because PFL mean widespread MSD with the addition of reduced functional ability [21]. The number of workers with PFL in this working population is relatively high, but the number of them recovering is even higher. These results reflect the tendency of PFL to fluctuate, a phenomenon previously described for MSD in various economic sectors [22,23]. Describing the course of upper-limb MSD for individual employees at an assembly-line factory, Aublet-Cuvelier et al. [12] showed important variations in individual clinical status, demonstrated by both a high annual appearance rate (13.5%) and a high annual recovery rate (44.3%). So PFL, which are a major health problem among the elderly because they decrease quality of life [24,25], also significantly affect younger people, in particular those still actively employed. There was an age effect but no gender difference in the PFL appearance rate in 1995. This last result is inconsistent with other population-based studies, which have shown that women have a greater risk of disability in early old age [26,27]. Our results may be explained by the specific nature of the population, which consisted of women who were still working in 1995. In other words, it is likely that the women with the most severe PFL in 1990 were no longer working in 1995 (healthy worker effect).

Physical occupational conditions appeared as stressors for PFL in both genders. Awkward and repetitive work may damage muscles, tendons and nerves and were a source of functional impairments [28,29]. Concerning non-standard hours, there is evidence that night shift workers experience adverse health effects [30]. These associations are close to those found for MSD, but our results show that the impacts of physical factors occur in less than 5 years and concern mainly new-onset exposure in both genders. Conversely, the lack of effect in the unexposed population in 1995 can be explained by the fact that the total duration may have been longer. Psychosocial working conditions appear to have a protective effect on PFL without an obvious Karasawek effect with job pressure (high psychological pressure), but high decision latitude and having means were associated with a decreased PFL appearance rate. Higher decision latitude and means give workers opportunities to apply and develop their skills and creativity and could prevent negative impacts on health [31,32]. Our results show that the extinction of these exposures between 1990 and 1995 had an adverse impact in less than 5 years, with an increase in PFL. Conversely, the new-onset high decision latitude exposure in 1995 compared to non-exposure in 1990 and 1995 did not have a significant effect. Implementing new psychosocial occupational conditions could mean changing the organization of work and, consequently, require more time. The PFL recovery rate was less related to physical and psychosocial factors in both genders, but mainly among women workers. Even if the PFL recovery rate was high, impacts of occupational conditions were low and changes to PFL could be related to multiple factors beyond the professional sphere. Some studies suggest that lifestyle characteristics have an impact on health parameters. Women in high and middle socio-economic classes are said to have higher stress levels because of the combined pressures of work and family life [33,34]. Interference of work and family life is more frequent among women and associated with long-term sick leaves among women who bear the main responsibility for housework and family life [35]. Changes in exposure to occupational factors between 1990 and 1995 were stratified by gender and were adjusted for age. The results were the same with emotional reactions and sporting activities.

Some limitations of our study must be pointed out. The data are 20 years old but seem relevant to the present period if we compare them with data from a recent survey carried out by the French department of labour [36]. So, in French industry 16.2% of workers declared an awkward work situation in 1984 compared to 34.2% in 2005, 33.7% declared repetitive work in 1984 compared to 36.4% in 2005, and 42.6% declared nonstandard hours in 1984 compared to 51.3% in 2005. Every worker in this sample was followed by an occupational physician, compared to about 75% of workers in the total French workforce. Nonetheless, we cannot guarantee that the sample is representative of the entire French working population. Participants lost between 1990 and 1995 belonged mainly to the cohort of 52-year-olds, and their absence may have biased the relationships between changes in PLFs and changes in working conditions. We chose the Nottingham Health Profile to estimate PLFs because it is one of the instruments most often used to assess subjective health in epidemiological surveys [18]. But this instrument uses dichotomous questions, which carries a risk of classification bias. Moreover, the assessments were based on participants’ subjective ratings. Self-reported function corresponds to objective measures of functional impairment [37], and we required the presence of at least two items to define the presence of PFL. Had we defined it using three items or more, the PFL rate would have been too low (less than 2.5% of the population). Most of the participants experiencing difficulties performing these activities were still working. This situation might seem surprising. Nonetheless, the three NHP items most often reported were: “I have difficulty standing up for any length of time,” “I have trouble leaning forward,” and “I have problems going up and down stairs.” In our population, these difficulties increased with age. Depending on the working conditions, work remains possible despite these difficulties. Moreover, some workers probably minimize their pain and disabilities during work. Conversely, no participants reported that they were totally unable to walk. Other working conditions, including repetitive work and the means to produce quality work, were also assessed using dichotomous questions and thus very sensitive to classification bias. Here, the validity of the information was checked by the occupational physicians, who verified the workers’ statements according to the job specifications included in the medical file. Almost 70% of the sample was examined in 1995 by the same occupational physician seen in 1990. The final limitation is that we have only a two-wave data set, and changes in working conditions and PFL were recorded simultaneously. Reversed causality may thus explain the associations found. The emergence of limitations at time 2 could affect the experience of working conditions at the same time. Nonetheless, the participants’ reports about their working conditions were checked by their occupational physicians, which would appear to limit this reverse causality risk. Finally, the data were collected only in 1990 and 1995, and no information was provided within this interval. Thus, transient cases that might have developed and disappeared between the scheduled examinations were not detected.

## Conclusion

The course of PFL is influenced by environmental constraints, especially at work. PFL do not represent a direct indicator of the severity of MSD, rather they are the result of the subject’s adaptation to changing events, especially in his or her work environment. The results of this study provide some suggestions for future preventive interventions by governments to keep people employed longer. In particular, workers without PFL may benefit from better-targeted preventive measures by maintaining protective psychosocial factors and avoiding exposure to physical stressors.

## Competing interests

The authors report no conflicts of interest.

## Authors’ contributions

MS, BC, FD provided the idea for the study. MS, BC, AL analyzed and interpreted the data. MS, AL, FD, CM, AT, BC wrote the paper. All authors revised it and approved the version to be published. MS is the guarantor. All authors read and approved the final manuscript.
